# A Constant-Force End-Effector With Online Force Adjustment for Robotic Ultrasonography

**DOI:** 10.1109/LRA.2021.3061329

**Published:** 2021-02-23

**Authors:** X. Bao, S. Wang, R. Housden, J. Hajnal, K. Rhode

**Affiliations:** 1 School of Biomedical Engineering and Imaging Sciences, King's College LondonKing's Health Partners, St Thomas' Hospital 150504 SE1 7EHLondonU.K.; 2 State Key Laboratory of Management and Control for Complex SystemsInstitute of Automation, Chinese Academy of Sciences 74522 Beijing100190China

**Keywords:** Robotic ultrasonography, online force adjustment, constant force, end-effector

## Abstract

In this letter, we propose a novel constant-force end-effector (CFEE) to address current limitations in robotic ultrasonography. The CFEE uses a parallel, motor-spring-based solution to precisely generate constant operating forces over a wide range and enable the ultrasound (US) probe to adapt to the abdominal contours autonomously. A displacement measurement unit was developed to realize the acquisition of probe position and precise control of the operating force. Moreover, the operating force can be adjusted online to maintain safety and continuity of operation. Simulations and experiments were carried out to evaluate the performance. Results show that the proposed CFEE can provide constant forces of 4-12 N with displacements of 0-8 mm. The maximum relative error of force generation is 8.28%, and the accuracy and precision for displacement measurement are 0.29 mm and ±0.16 mm, respectively. Various operating forces can be adjusted online during the same operation. Ultrasound images acquired by the proposed CFEE are of equally good quality compared to a manual sonographer scan. The proposed CFEE would have potential further medical applications.

## I. Introduction

Ultrasonography is of increasing importance in recent years and has proliferated with examples being fetal, abdominal, vascular and cardiac ultrasound (US) imaging [1]. However, sonographers are prone to suffer from strain injuries with prolonged operation time and repeat procedures [2]. Moreover, risks of X-rays exist when sonographers perform the intraoperative ultrasonography during interventions, such as cardiovascular interventions [3]. Radiation-shielded garments can partly reduce the radiation risks but result in cervical and lumbar spondylopathy because of their weight. Thus, robotic ultrasonography has potential applications in healthcare due to its precision, dexterity, and repeatability [4]. Besides, robotic ultrasonography enables sonographers to perform ultrasound examinations remotely especially for underserviced rural and remote communities [5].

In extracorporeal robotic ultrasonography, a robotic arm is commonly used to hold a commercial US probe and moves it along the surface of the skin for ultrasonic image acquisition. In the interaction between the US probe and skin, the contact force that the US probe applies to the tissue is critical for safe operation and robust image acquisition. Excessive contact forces could be uncomfortable for patients and cause potential injuries, while inadequate contact forces may result in low-quality images. Thus, much research has been devoted to contact force control and safety management of extracorporeal robotic ultrasonography. In [6] and [7], a US robot was proposed to perform fetal and abdominal US examinations. A robotic arm grasps the US probe with a probe holder and keeps it in contact with the patient's skin. The expert can apply additional pressure limited to 15 N when pushing on the probe. Smith-Guerin *et al.* developed an expert-patient mobile tele-echography system to control the US probe remotely and reproduce the motion of a US probe held by the expert [8]. The contact force can be controlled based on expert experience and a maximum allowable threshold. Tsumura *et al.* proposed a US robot to maintain the contact force in a certain range and passively adjust the US probe posture relative to the body surface by using a linear actuator and constant spring [9]. These reported robots maintain the contact force through precise control of the robotic arm with sensors and actuators. They can only limit the control force within a certain range, but cannot precisely control and adjust the operating forces online based on the clinic requirements. Moreover, these methods do not separate the contact force control from the robotic arm control, which is not conducive for the control and function expansion of the robotic arm. The development of a special end-effector is necessary for individual control of the contact force and movement: the robotic arm controls the movement of the US probe and the end-effector controls the contact force of the US probe. The individual control method enables the robotic arm to be controlled in a more straightforward manner with higher response accuracy since the robotic arm only needs to focus on position adjustment. Meanwhile, the end-effector would improve the force control accuracy by the individual control method. High force control accuracy results in high-quality images and safer operation. In our previous research [10], to achieve the individual control of the force and motion, we investigated a customized spring-loaded ball clutch joint to limit the maximum force applied to the US probe. This clutch joint is integrated with a robotic arm. However, it cannot provide a constant operating force, but only a limited range of force, which needs to be set before the operation and cannot be adjusted online.

To obtain constant operating forces, various constant-force mechanisms were investigated and applied in different fields. [11] proposed a constant-force mechanism, which generates force using two sliders and springs. This mechanism could be used as additional accessories to prevent damage to the parts due to errors in macro positioning. Lambert *et al.* presented a constant-force mechanism using a 11-revolute joints spatial linkage using simple pin joints and two regular springs [12]. Passive linear sliders with ball bearings are not suitable for use in the medical environment or miniaturization, while PTFE pin joints can be made smaller than ball bearings and are bio-compatible but may result in low quality of the output force. Liu *et al.* developed a constant-force mechanism that uses a curved surface to generate the force [13]. The designed mechanism can produce a constant output force of 300 N, but the shape of the curved surface needs to be optimized according to a particular operating force. In [14], a compliant constant-force gripper is proposed to realize constant force control by using the buckled fixed-guided beam. It is able to work in a 530 mN constant-force range up to 220 μm. However, these related studies are not suitable for use in the extracorporeal robotic ultrasonography due to the mismatched working range, lack of force adjustment online, and difficulty in high precision control.

In order to improve the safety of robotic ultrasonography, the US probe needs to be able to adapt to different scanning contours, such as the abdomen of pregnant women, which varies substantially with the individual and the number of weeks of gestation. Huang *et al.* used a depth camera to capture the point cloud of the skin surface and the scan range and path for the US probe was obtained to control the robot arm [15]. This type of method allows the US probe to adapt to the contours of scanned areas well but results in a complicated system.

In this research, to overcome the above-mentioned limitations, we propose a novel constant-force end-effector (CFEE), which can provide a constant operating force over a wide range and adjust the operating force online to adapt to various conditions and the contours of scanned areas. The main contributions of this research are as follows:

1) A novel parallel, motor-spring-based solution was proposed to generate constant operating force over a wide range. The constant operating force enables robotic ultrasonography to be performed safely and improves comfort for patients. Besides, with the operating force adjusted online during the scanning, the continuity of operations is increased.
2) We proposed a displacement measurement unit to realize the acquisition of the US probe position and precise control of the operating force. The US probe can move freely up and down in a broader range, and thus it can automatically adapt to the contours of scanned areas. Moreover, sonographers do not need to pay attention to the value of the operating force and the contours of scanned areas. Therefore, this method reduces the workload of sonographers and improves operational efficiency.
3) This end-effector is independent of the robotic arm and performs autonomous control, which is conducive to the expansion of other functions of the robotic arm. It has a simple structure and low cost and can be used in other medical applications.

The remainder of this letter is organized as follows. The clinical requirements and design details of the proposed end-effector are described in Section II. Section III presents the experimental set-up for performance evaluation, as well as the results and discussions. Finally, the conclusion is given in Section IV.

## II. Method

### A. Clinical Data Acquisition Requirements

In our previous research, we investigated the operation requirement through fetal US scanning by using a standard US probe and force/position measurement instruments. A US probe (X6-1, Philips, NL) is located in a bespoke probe holder (detailed in [16]), which incorporates a six-axis force sensor (Nano 17, ATI, USA) and an electromagnetic tracking sensor (Aurora, NDI, CA). The six-axis force sensor captures the operating force and torque during scanning while the electromagnetic tracking sensor measures the position and orientation of the probe. A US scanner (EPIQ7, Philips, NL) is connected to the standard US probe. Trained sonographers performed the fetal US scanning on pregnant women between 18 to 24 weeks of gestation at St Thomas’ Hospital, London, U.K. (Study title: Intelligent Fetal Imaging and Diagnosis (iFIND)-2: Further US and MR Imaging, Study reference: 14/LO/1806). The force applied to the US probe face and the position of the US probe face were captured. The data of six women were analyzed by extracting time ranges during which standard fetal anomaly views were imaged. The mean value and standard deviation of the axial force and displacement are 4.72 ± 0.42 N and 7.33 ± 0.72 mm, respectively. Based on the 95th percentile calculation, the US probe needs to withstand an axial force of 8.01 N and an axial displacement of 5.22 mm to obtain no significant deterioration of the imaging view [17].

### B. Overall System

**Fig. 1.**
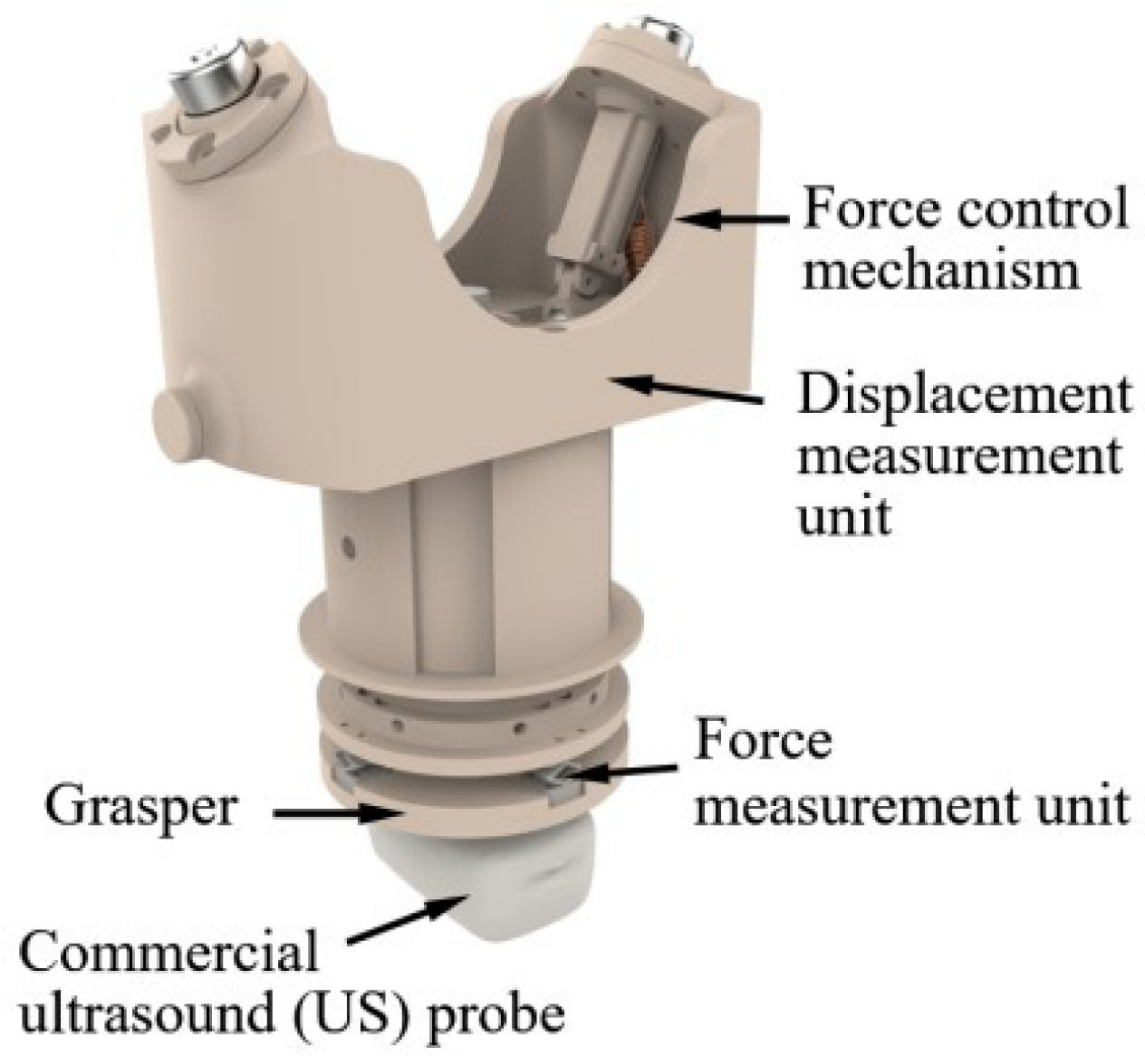
Diagram of the proposed constant-force end-effector (CFEE).

The CFEE consists of a force control mechanism, displacement measurement unit, force measurement unit and grasper (shown in Fig. 1). The force control mechanism provides the necessary contact force and keeps it in a suitable range during the scanning. The displacement measurement unit measures the displacement of the US probe in the axial direction. The contact force during the scanning is captured by the force measurement unit and can be used as force feedback or early warning. The grasper holds a US probe and enables it to move along the abdomen with the CFEE.

**Fig. 2.**
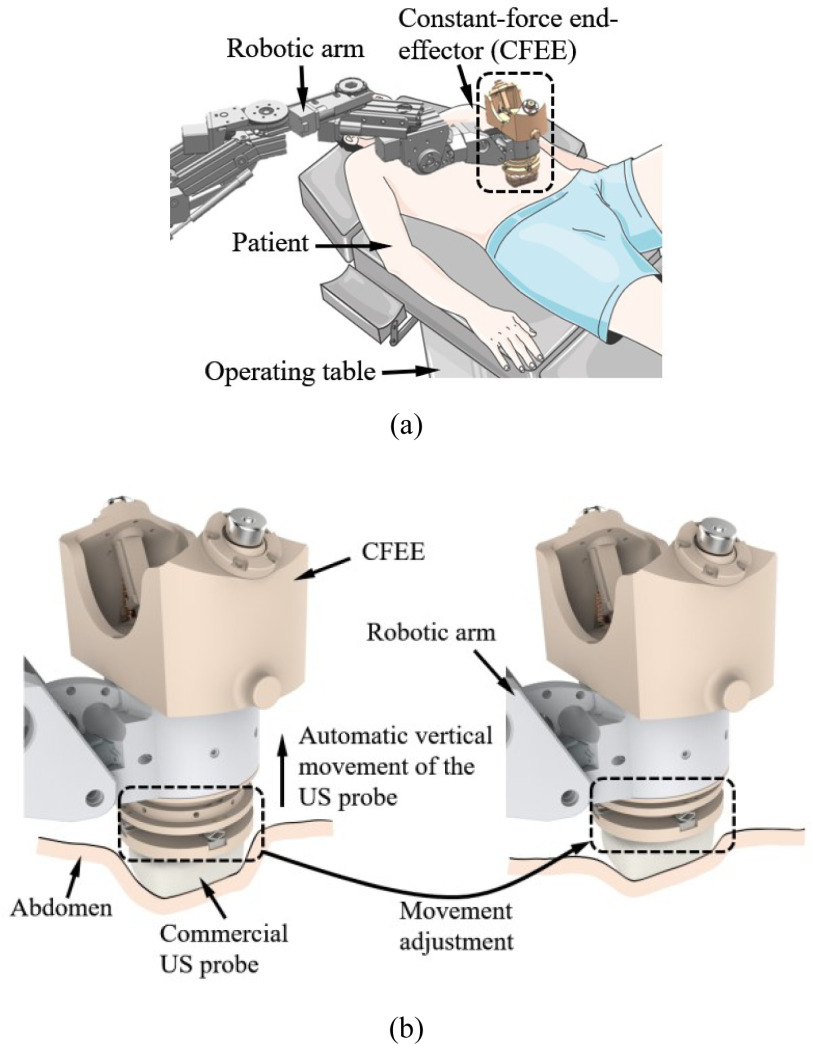
(a) CFEE performing the ultrasound (US) scanning; (b) workflow of the CFEE with automatic adjustment.

The CFEE is mounted on the end of a robotic arm, and the robotic arm can be teleoperated by sonographers. During the scanning, sonographers operate the robotic arm and let it move to a target position to obtain the required imaging view. In our previous US robot [18], when the end-effector is placed in the area of the target view using the robotic arm, the commercial US probe moves vertically to the target. The contact force between the abdomen of pregnant women and the US probe increases immediately with the vertical movement. Since the end-effector is teleoperated, the sonographer cannot accurately determine the contact force. The uncontrolled contact force not only affects the acquisition of US images but also causes safety concerns. When this proposed CFEE is used to replace the traditional end-effector, i.e., simple probe holder, the US probe can adjust its movement in the vertical direction according to the contact force threshold set before the scanning or online (Fig. 2). Before performing the scanning, the CFEE is placed in roughly the right place above the abdomen with the movement of the robotic arm. Then the CFEE enables the US probe to move automatically in the vertical direction with a constant operating force and autonomously adapt to the abdominal contours (Fig. 2(b)). Therefore, sonographers are able to concentrate all efforts on target positioning and image acquisition and do not need to pay attention to the operating forces and abdominal contours, which improves the operation safety and efficiency.

### C. Force Control Mechanism

#### 1) Structure

The conventional hinged-lever constant-force mechanism uses a spring to generate force, and it has obvious application limitations, such as small workspace and payload, inaccuracy forces, and forces that cannot be adjusted online [19]. In this research, we propose a novel parallel, motor-spring-based solution which can address these challenges.

**Fig. 3.**
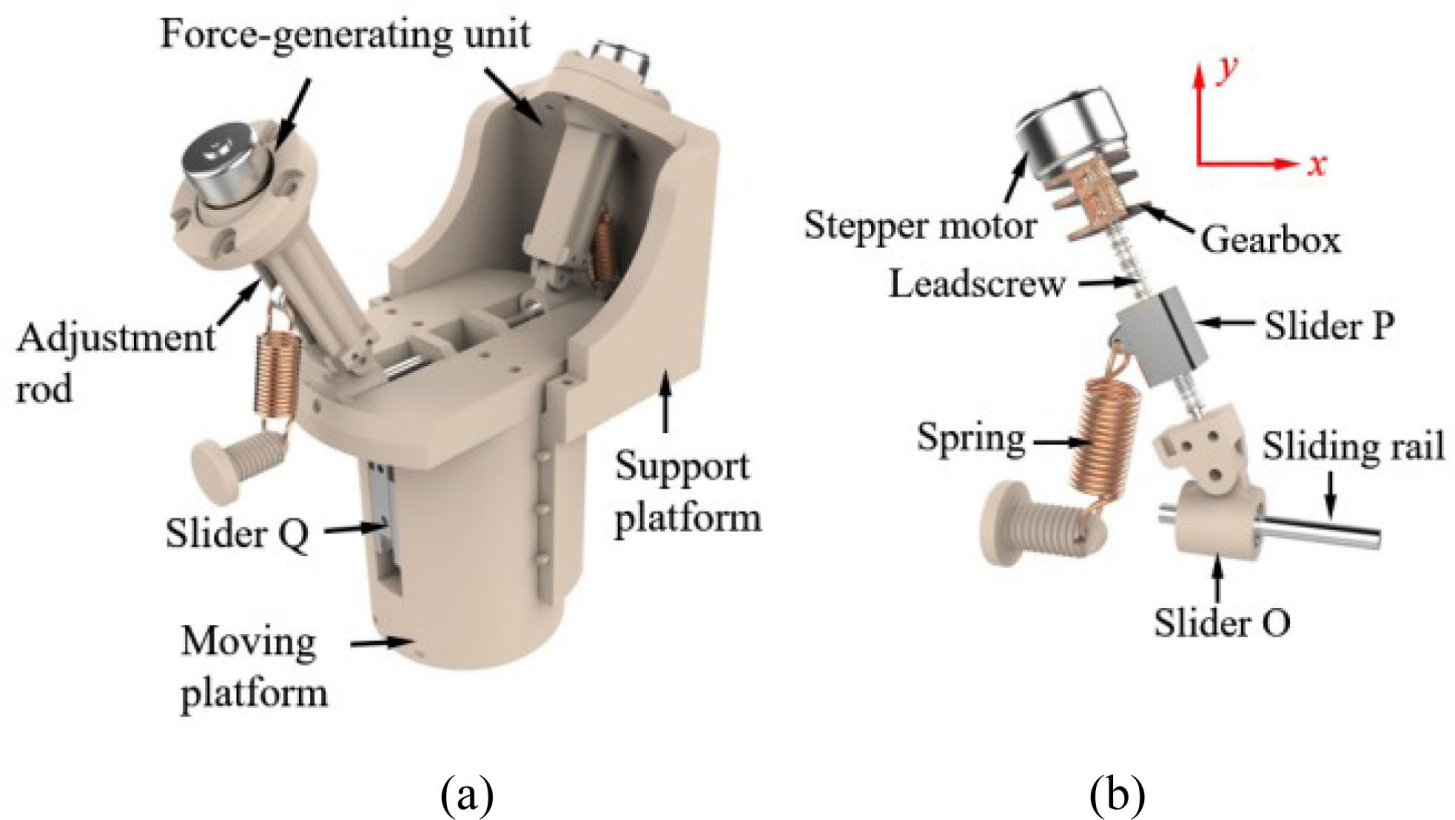
(a) Partial structure of the CFEE; (b) internal structure of the force-generating unit.

As shown in Fig. 3(a), the force control mechanism is composed of a support platform, two force-generating units and a moving platform. The support platform is connected to a robotic arm and provides support to all components of the CFEE. Two force-generating units are connected in parallel and linked with the moving platform. The force-generating unit generates the force through the cooperation of the spring and adjustment rod and then transmits it to the US probe through the moving platform. Fig. 3(b) shows the internal structure of the force-generating unit. Slider O moving on the sliding rail keeps the generated force in the *y*-direction and eliminates the component in the *x*-direction. Slider P connected with the spring works with the leadscrew. A gearbox is used to transfer the torque from the stepper motor to the lead screw. Thus, the adjustment rod can change the position of the slider P in the axial direction by using the stepper motor. Changes in the position of the slider P result in different forces generated by the force generating unit. We use this principle to generate a force accurately and adjust the force automatically online. As shown in Fig. 3(a), the moving platform linked with the sliding rail is mounted on slider Q, and thus it can move only in the *y*-direction. Since the moving platform provides support to the force measurement unit and grasper, it ensures the transmission of the generated force and the measurement of the contact force during the scanning. In this design, a gearbox with a reduction rate of 20:1 was used to link to a stepper motor (HBD12-D, MCRT, CN). A tension spring (DE675, Accurate, JP) with a stiffness of 1.236 N/mm was adopted to generate tension for the moving platform. A rail kit (BSP1025SL, IKO, JP) acts as slider Q and a bearing (C-LMUM3, MISUMI, JP) worked as the internal sliding part of slider O.

Compared to using a single force-generating unit, two units in parallel double the generated force. A generated force with great value requires a longer adjustment rod (link AD), high stiffness springs, and higher-power motors, and thus these requirements will result in a CFEE with a larger volume. Moreover, the symmetrical configuration can eliminate the horizontal force component and increase the stability of operations.

#### 2) Kinematics Modelling and Force Analysis

**Fig. 4.**
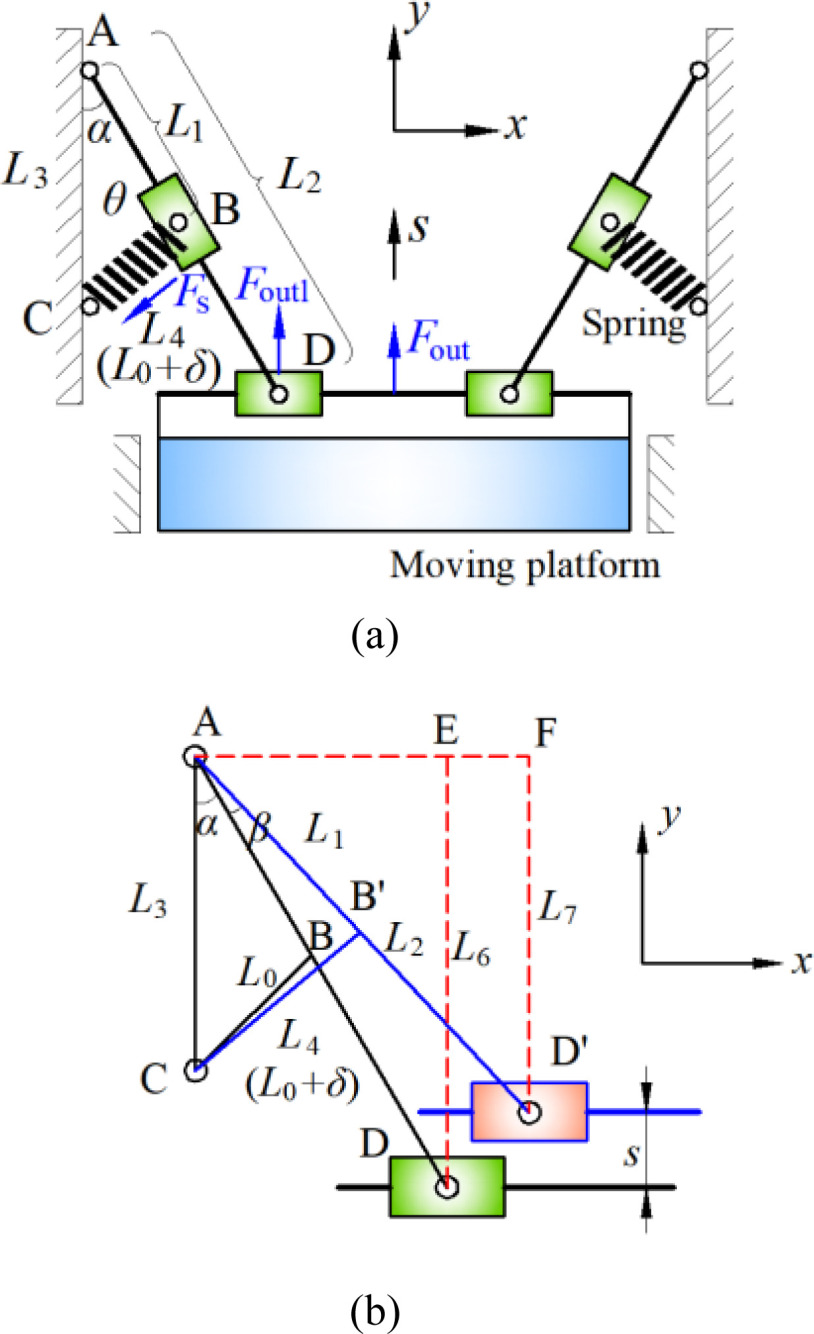
(a) Kinematic diagram of the force control mechanism; (b) kinematic workflow of the force control mechanism.

In order to determine the force generated by the CFEE under the US probe displacement variation and spring deformation, a mathematical model is established in this section and used to predict and control the generated force during the scanning. A kinematic diagram of the force control mechanism is shown in Fig. 4(a). The two force-generating units have a similar performance because of the same structures and parallel connections. Thus, we first analyze one of them separately and then investigate the overall performance by considering the combination.

When the US probe contacts and presses the abdomen to capture the US image, the required contact force for the data acquisition is provided by the CFEE, and it is the output of two force-generating units. Taking point A as the fulcrum, a balance equation is written as:

\begin{equation*}
{F_{{\rm{outl}}}}{L_2}\sin \alpha - {F_{\rm{s}}}{L_1}\ \sin \theta = {\rm{\ }}0\tag{1}
\end{equation*}

where ${F_{{\rm{outl}}}}$ is the output of the force-generating unit, ${L_2}$ represents the length of the adjustment rod (link AD), $\alpha $ is the angle formed by the link AD and the support platform (link AC), ${F_{\rm{s}}}$ is the force generated by the spring, ${L_1}$ represents the distance between point A and point B, and$\ \theta $ is the angle formed by the spring (line BC) and link AD. According to the law of sines in triangle ABC, we derive a relationship equation:

\begin{equation*}
\frac{{{L_3}}}{{\sin \theta }} = \frac{{{L_4}}}{{\sin \alpha }} \tag{2}
\end{equation*}

where ${L_3}$ represents the length of link AC, and ${L_4}$ is the length of line BC. Substituting (2) into (1) results in

\begin{equation*}
\ {F_{{\rm{outl}}}} = \ {F_{\rm{s}}}\frac{{{L_1}{L_3}}}{{{L_2}{L_4}}}.\tag{3}
\end{equation*}

Since the spring is an extension spring, the equations describing the relationships in the spring is as follows:

\begin{equation*}
\left[ {\begin{array}{rcl} {{F_{\rm{s}}}}\\ {{L_4}} \end{array}} \right] = \left[ {\begin{array}{rcl} {{F_0}}&{k\delta }\\ {{L_0}}&\delta \end{array}} \right]\ \left[ {\begin{array}{rcl} 1\\ 1 \end{array}} \right]\tag{4}
\end{equation*}

where ${F_0}$ is the initial tension force of the spring, $k$ represents the stiffness of the spring, $\delta $ is the extension of the spring, and ${L_0}$ represents the initial length of the spring without external loading. Substituting (4) into (3) results in

\begin{equation*}
{F_{{\rm{outl}}}} = \ k\frac{{{L_1}{L_3}}}{{{L_2}}} + \frac{{{L_1}{L_3}}}{{{L_2}}} \cdot \frac{{{F_0} - k{L_0}}}{{{L_0} + \delta }}.\tag{5}
\end{equation*}

According to (5), the output of the force-generating unit depends on the dimension parameters of links and characteristics of the spring. During the scanning, the extension of the spring changes with the displacement of the US probe in the y-direction. To obtain the relationship between them, we draw a kinematic workflow based on their movement characteristics (Fig. 4(b)). When the US probe needs to work with a greater depth relative to the skin surface, the slider Q moves in the +y-direction. The slider Q moves from point D to point D′ and point B moves to point B′ with the spring extension. Since the US probe has the same displacement with slider Q in the y-direction, the displacement of the US probe can be obtained by

\begin{equation*}
s\ = {L_6}\ - {L_7}\tag{6}
\end{equation*}

where ${L_6}$ represents the length of line DE and ${L_7}$ represents the length of line D′F. According to the geometry of triangles ADE and AD′F, we can obtain:

\begin{equation*}
\left[ {\begin{array}{rcl} {{L_6}}\\ {{L_7}} \end{array}} \right] = \left[ {\begin{array}{rcl} {{L_2}\cos \alpha }\\ {{L_2}\cos \left({\alpha + \beta } \right)} \end{array}} \right]\tag{7}
\end{equation*}

where $\beta $ is the angle formed by link AD and AD′. Based on the law of cosines in triangles ABC and AB′C, the extension of the spring can be derived by substituting (7) into (6):

\begin{equation*}
\delta \ = \sqrt {L_0^2 + 2{L_1}{L_3}s/{L_2}} {\rm{\ }} - {L_0}\tag{8}
\end{equation*}

Therefore, when (8) is substituted into (5), the relationship between the force-generating unit and the displacement of the US probe can be written as:

\begin{equation*}
{F_{{\rm{outl}}}} = {\rm{\ }}k\frac{{{L_1}{L_3}}}{{{L_2}}} + \frac{{{L_1}{L_3}}}{{{L_2}}} \cdot \frac{{{F_0} - k{L_0}}}{{\sqrt {L_0^2 + 2{L_1}{L_3}s/{L_2}} }}\tag{9}
\end{equation*}

If the extension spring has a force-displacement curve starting from the origin, the initial tension force of the spring ${F_0}$ can be expressed as $k{L_0}$ and thus the output of the force-generating unit can be written as ${F_{{\rm{outl}}}} = \ k\frac{{{L_1}{L_3}}}{{{L_2}}}$. The output force-generating unit is constant and does not change with the displacement of the US probe. However, the initial tension force exists inevitably and thus, the output of the force-generating unit changes with the displacement of the US probe. To adjust its value online (keeping the force constant or changing the force in some specific scanning site) based on the clinical requirement during the scanning, we take ${L_1}$ as the control parameter and ${L_1}$ can be obtained by (8) and (9).

Since the force-generating units are connected in parallel, the output of the force control mechanism can be obtained by

\begin{equation*}
{F_{{\rm{out}}}} = \ 2{F_{{\rm{outl}}}}.\tag{10}
\end{equation*}

The displacement measurement unit measures the displacement of the US probe and sends signals to the control system. The control system controls the stepper motor to change ${L_1}$ according to (8)–(10) and then adjusts the generated force online. Since changes in operating velocity during the scanning occur slowly, the acceleration is not significant, and we will not discuss the dynamics in this letter.

### D. Displacement and Force Measurement

**Fig. 5.**
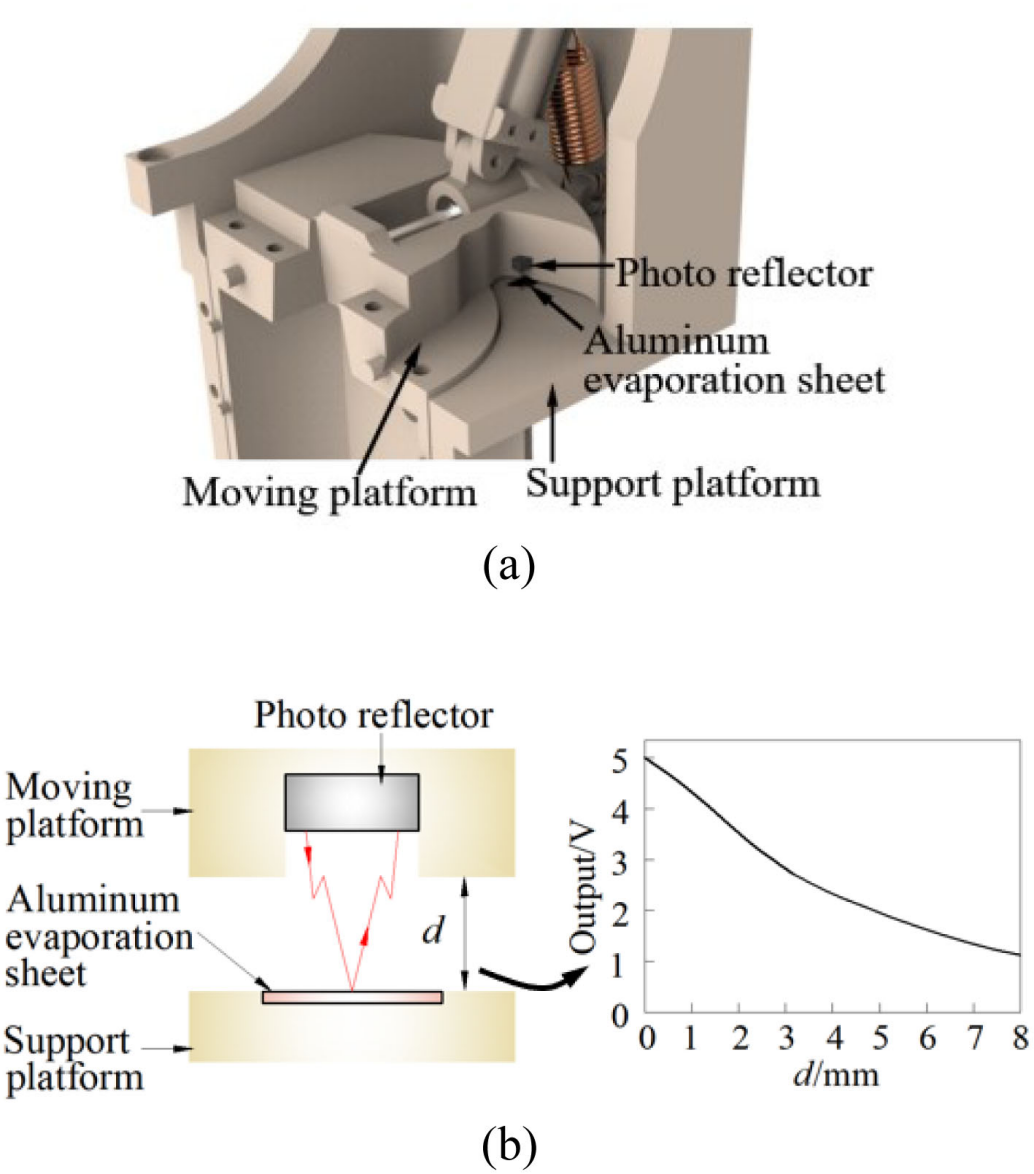
(a) Installation diagram of the displacement measurement unit; (b) working principle of the displacement measurement.

To automatically adjust the generated force online, we develop a displacement measurement unit to capture the movement of the US probe during the scanning. Considering the measurement accuracy, the size and cost of the sensing unit, we choose the photoelectric reflection measurement principle. A photo reflector, (NJL5909RL-4, JRC, JP), 1.9 mm × 2.65 mm × 1.6 mm, is used and integrated into the developed CFEE. As shown in Fig. 5(a), the photo reflector is set on the moving platform and an aluminium evaporation sheet, used to reflect the light from the photo reflector, is mounted on the support platform. Fig. 5(b) shows the working principle of the displacement measurement and the relationship between the distance and output. This photo reflector consists of a LED and photo-transistor. When the photo reflector moves close to the support platform, the infrared signal from LED is reflected at the aluminium surface and collected by the photo-transistor. Changing the distance between the photo reflector and aluminium evaporation sheet results in varying reflected light intensity. The photo reflector outputs voltage based on the light intensity variation.

**Fig. 6.**
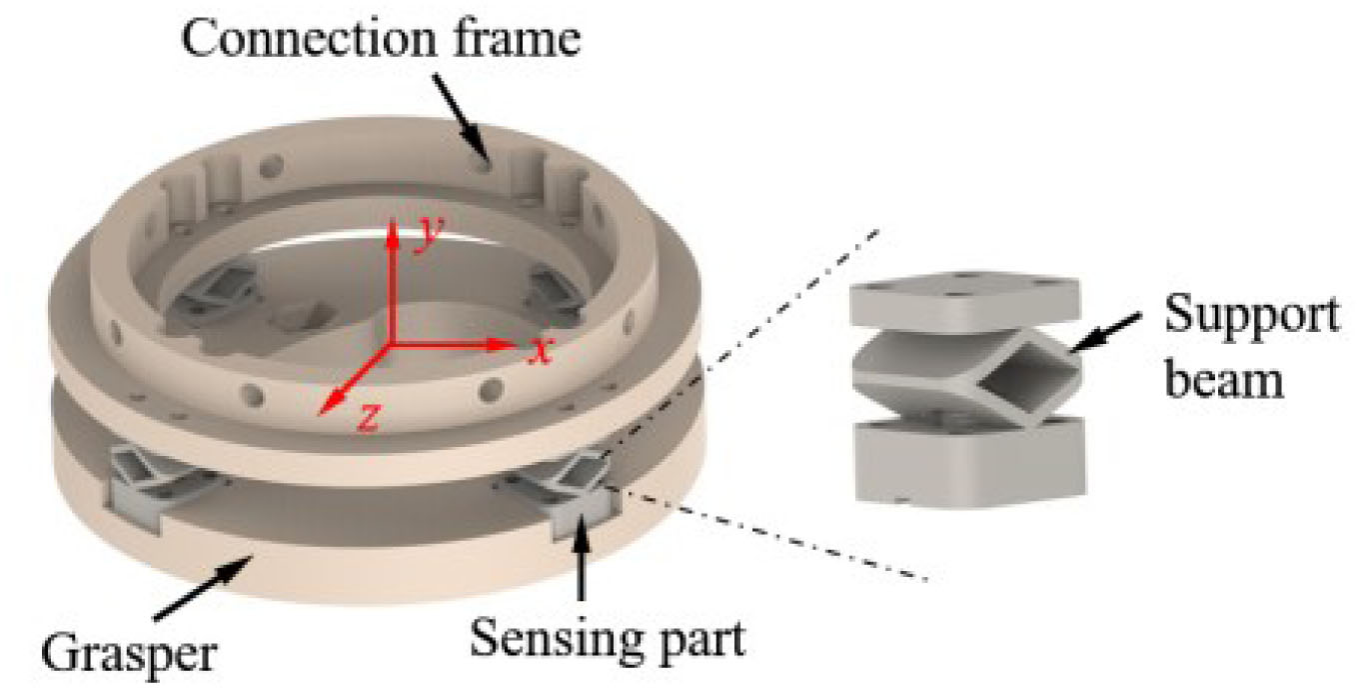
Overview of the force measurement unit.

The overview of the force measurement unit is shown in Fig. 6. It consists of a connection frame and four sensing parts. Four sensing parts evenly arranged in a ring connect the connection frame with the grasper. A photointerrupter is set in the sensing part to measure the deformation of the support beam when an external load is applied. The force in the *y*-direction and torques around *x* and *z* directions can be calculated during operations based on the deformation.

### E. Compliance Design

**Fig. 7.**
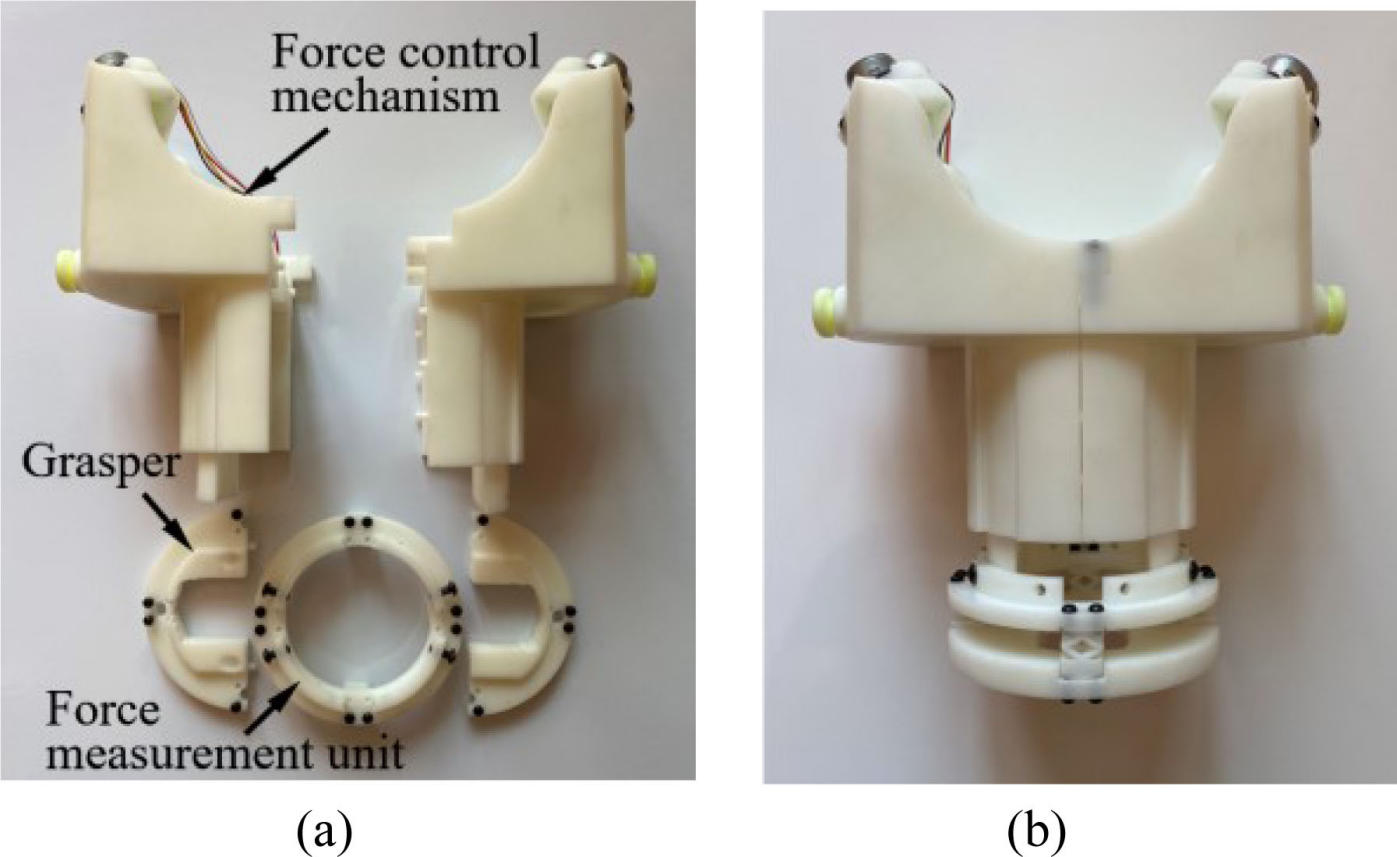
(a) CFEE divided into parts; (b) assembled CFEE.

The US probes used in ultrasonic detection have different shapes and sizes. To enable the CFEE to work with any shapes of US probes, we design the grasper as a replaceable component. The internal shape of the grasper should be the same as the external shape of the US probe, and thus the grasper can hold the probe firmly. The US probe is first scanned with a CT scanner and then its 3D mesh is extracted and imported into CAD software. A customized grasper can be designed based on this 3D model. In order to facilitate installation and disassembly of the US probe, especially for wired US probes, whose wires need to go through the CFEE and robotic arm, the CFEE is developed as a removable two-piece structure. The two-piece design would solve cable-assembly issues. As shown in Fig. 7, most components of the CFEE, including the moving platform, support platform, and grasper, are divided into two pieces. This provides excellent flexibility in customized CFEE design.

### F. Prototype

The prototype of the proposed CFEE is shown in Fig. 7. The proposed CFEE has the dimensions of 140 mm × 70 mm × 168 mm. In this research, the overall size of the CFEE is mainly designed to be applied to various types of US probes, especially wired US probes. The height will be reduced accordingly for the design of probes with smaller lengths. The proposed CFEE weighs 218 g and thus it can be easily integrated into the robotic arm. Besides, the stepper motors are controlled by motor drivers (TB6600 upgraded, JXINW, CN), and the analog signals of the displacement unit are collected through a data acquisition module (DAM-3918, ART, CN) and transformed to a PC through RS485. All programs are running on Microsoft Visual C++.

## III. Experimental Validation and Discussion

### A. Displacement Measurement

**Fig. 8.**
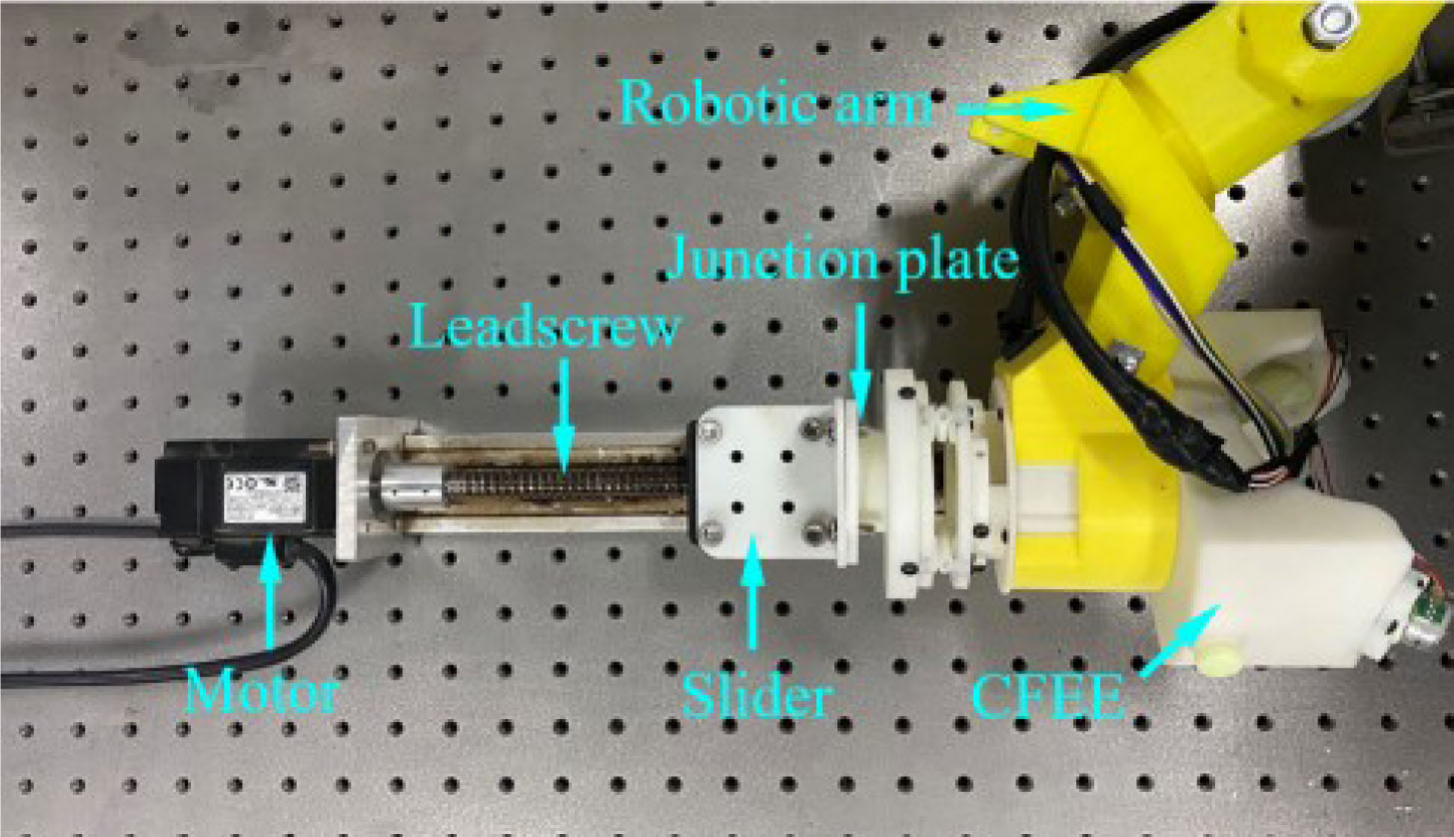
Experimental set-up for the evaluation of the displacement measurement unit.

To verify the performance of the displacement measurement unit, accuracy and precision evaluation experiments were carried out. As shown in Fig. 8, a junction plate mounted on the slider is connected with the grasper of the CFEE. The slider can move forwards and backwards with the rotation of the high-precision leadscrew (motor). Thus, the movement of the moving platform can be adjusted by the control of the motor. In the accuracy evaluation experiments, the moving platform advanced in 0.5-mm increments over a range of 8 mm at a speed of 3 mm/s. The displacement measurement unit obtained the positional data at each increment. These operations were repeated nine times. In the precision evaluation experiments, the moving platform advanced to the 4-mm position nine times, and the standard deviation of the error in the measured position was calculated.

The measured accuracy and precision of the displacement measurement unit are 0.29 mm and ±0.16 mm, respectively. The performance evaluation shows the ability of the CFEE to measure the displacement of the US probe (moving platform). This measurement accuracy and precision affect the force generation performance and the performance evaluation is detailed in Section III-C.

### B. Force-Motion Workspace

To evaluate the attainable forces and motions of the CFEE to satisfy the imposed clinical requirements for the US probe motion, we investigated the range of the generated force and motion and the relationship between them. The generated forces, US probe displacements and control parameter (${L_1}$) were explored and a diagram of force-motion workspace was obtained by using MATLAB based on (8)–(10). The range of the US probe displacement was first calculated, and then the results and the range of *L*_1_ were used as independent variables to calculate the generated force. The spring specification regarding this simulation contains three elements: *k* = 1.236 N/mm, *F*_0_ = 6.257 N, and *L*_0_ = 31.9 mm. The lengths of the links are as follows: 9 mm ≤ *L*_1_ ≤ 35 mm, *L*_2_ = 45 mm, and *L*_3_ = 40 mm. During the calculation, the lengths of link AB, AC, and BC were set to meet the triangle inequality ensuring the existence of triangle ABC. Additionally, the data space of the control parameter *L*_1_ was obtained by taking the US probe displacement and generated force as independent variables.

**Fig. 9.**
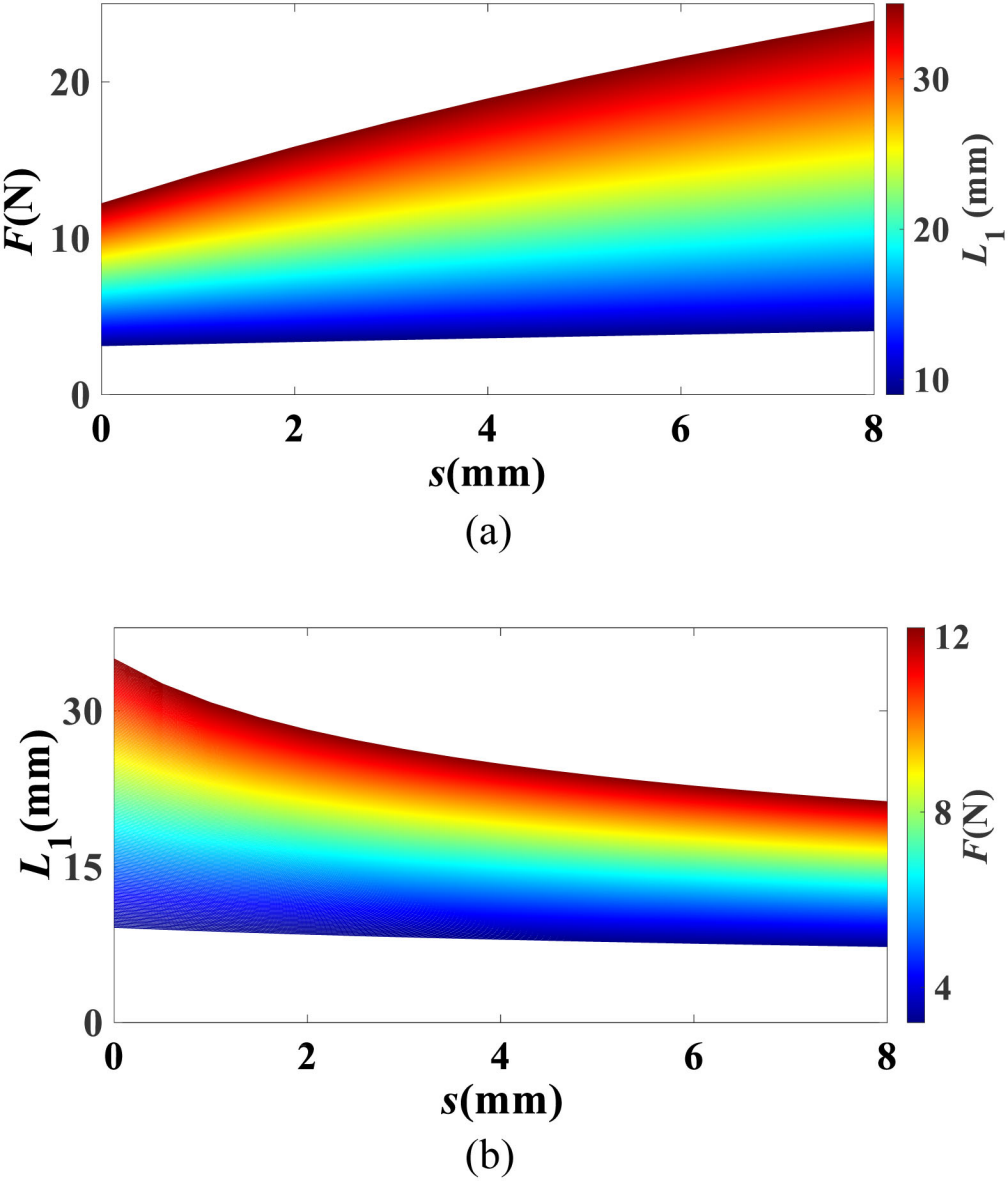
(a) Force-motion workspace of the proposed CFEE; (b) the data space of the control parameter ${L_1}$.

The diagram of the force-motion workspace is shown in Fig. 9(a). The CFEE can provide forces of 3 N to 12 N with the US probe displacement of 0 mm, and forces of 4 N to 24 N with the US probe displacement of 8 mm. The available workspace of the force and motion for the US probe is the colour zone in Fig. 9(a). Therefore, based on the analysis in Section II-A, the proposed CFEE satisfies clinical data acquisition requirements in scanning (axial force of 8.01 N and axial displacement of 5.22mm). Moreover, the data space of the control parameter ${L_1}$ was calculated and shown in Fig. 9(b). The value of ${L_1}$ can be selected according to this figure when a force will be generated with different values and with various displacements of the US probe. In other words, on the one hand, a generated force can be kept constant by changing the value of ${L_1}$. On the other hand, constant force operations that require different operating forces can be realized based on the adjustment online of ${L_1}$. The proposed CFEE was developed based on the fetal US scanning data of six pregnant women, and the maximal required force and motion may vary with some pregnant women with special physiques. Moreover, great attainable force and motion (e.g., more than 8 mm and 12 N) would be required for some other US inspections. Even so, the proposed CFEE is also feasible by redesigning the parameters of related components, such as lengths of links, stiffness of springs, etc. The great generated force and motion would be obtained by changing the components but result in the CFEE with a larger dimension.

### C. Force Generation

In order to evaluate the impact of displacement feedback on the force generation and measure the accuracy of the force generation, we investigated the performance with and without the displacement feedback provided by the displacement measurement unit.

**Fig. 10.**
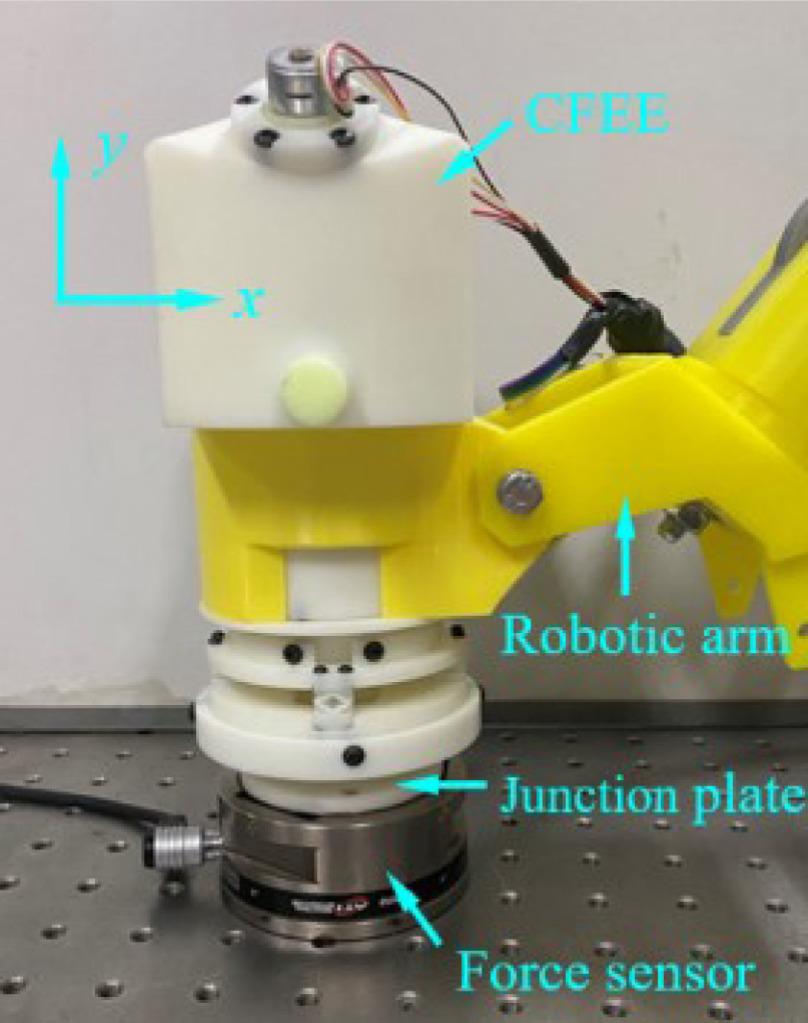
Experimental set-up for the force generation testing.

In the experiments without using displacement feedback, the performance of the generated force was investigated through simulations with MATLAB. Simulation parameters were the same as those in Section III-B. The generated forces with different values were calculated and compared to the expected ones (4 N, 6 N, 8 N, 10N, and 12 N). The maximum errors and relative errors were then obtained. In the experiments using the displacement feedback, we measured the generated forces with various values set before and compared them to expected ones. The experimental setup is shown in Fig. 10. A force sensor (Gamma, ATI Industrial Automation, Inc., USA) connects with the CFEE through a junction plate. We set the CFEE to generate forces of 4 N, 6 N, 8 N, 10 N, and 12 N, in turn. We pushed the CFEE by the robotic arm within the rated workspace of the CFEE (8mm) and the force with the set value was generated and then measured by the force sensor. During this process, displacements were measured by the displacement measurement unit and used to control the motors. To simulate the procedures of the real US scanning, the operations were performed randomly. Every operation was repeated nine times and errors were calculated.

Table I and II show errors of the generated forces with and without the displacement feedback. In the simulations without the displacement feedback, all errors increase dramatically with the increase of the set forces. This is due to the working characteristics of the CFEE. The maximum error is 11.34 N, and the maximum relative error is 94.48%. The CFEE cannot meet the clinical requirements without using the displacement feedback. In the actual test experiments, the errors are obviously reduced with the displacement feedback. The maximum relative error is 8.28%. Average relative errors seem to have slightly larger values with the increase of the expected force. We think it correlates with the shape of the data space of the control parameter ${L_1}$ (shown in Fig. 9(b)). A great generated force requires a larger adjustment range of control parameter ${L_1}$ with the same displacement change. The larger adjustment range of ${L_1}$ results in more moving time for slider P to get to the expected position. Increasing the moving speed of slider P (using a high-speed motor or gearbox) will decrease relative errors, but great generated force would also have bad performance than the small generated force due to the characteristic of the proposed device (i.e., the shape of the data space of ${L_1}$).

**TABLE I:** Errors in the Simulations Without the Displacement Feedback

Expected force (N)	*L*_1_ (mm)	Maximum error (N)	Average error (N)	Maximum relative error	Average relative error
4	11.47	1.52	0.79	37.97%	19.68%
6	17.21	3.25	1.71	54.19%	28.55%
8	22.94	5.50	2.94	68.80%	36.76%
10	28.68	8.22	4.45	82.24%	44.54%
12	34.41	11.34	6.22	94.48%	51.80%

**TABLE II:** Errors in the Actual Test Experiments Using the Displacement Feedback

Expected force (N)	Maximum error (N)	Average error (N)	Maximum relative error	Average relative error
4	0.33	0.18	8.25%	4.50%
6	0.45	0.31	7.50%	5.17%
8	0.58	0.42	7.25%	5.25%
10	0.77	0.62	7.70%	6.20%
12	0.98	0.81	8.17%	6.75%

### D. Contact Experiment

In this section, we verified the performance of the CFEE to keep the operating force constant and to adjust the operating force online while maintaining adaptation to various abdominal contours. The experimental setup is the same as that in the previous experiment shown in Fig. 10. The CFEE was set to output a constant operating force before experiments. An operator pressed the force sensor randomly in the - y-direction to simulate possible changes in abdominal shape during the scanning. The actual operating forces were measured by the force sensor during these operations. Two experiments with different constant operating forces were carried out. The constant operating forces for the two experiments were set as 6 N and 8 N, respectively.

In order to verify the performance of the CFEE to adjust the operating force online, we changed different constant operating forces during operations. Unlike the previous settings, the CFEE was set to output 6 N, 8 N, and 6 N, in turn, during the same operation.

**Fig. 11.**
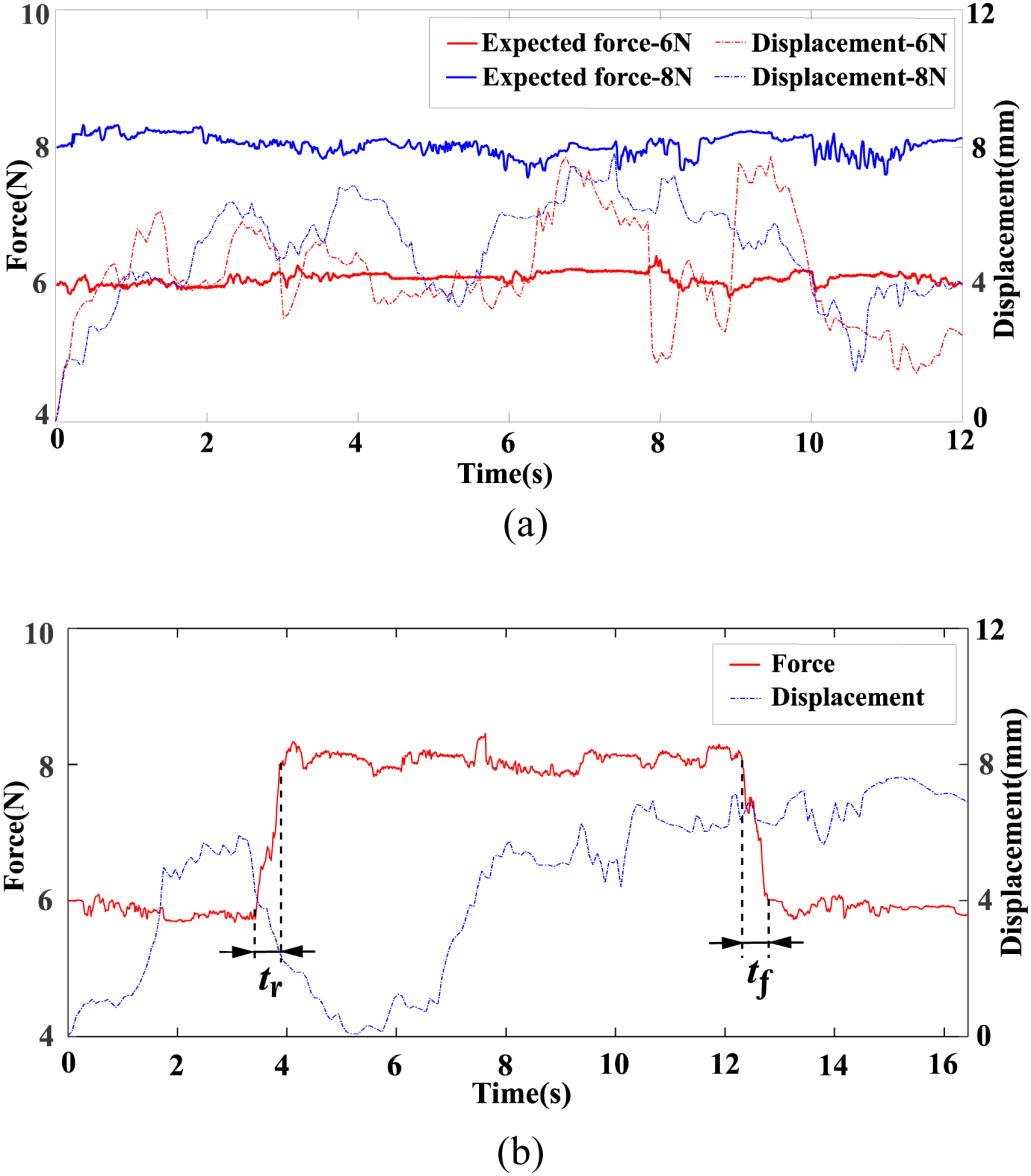
Results of the contact experiments: (a) two operations with two different operating forces; (b) one operation with operating force adjustment online.

Results of the two experiments with different constant operating forces were merged and shown in Fig. 11(a). The measured forces fluctuate slightly relative to the expected forces. Fig. 11(b) shows the results of the online adjustment of the operating forces. The operating force was adjusted from 6 N to 8 N and then was changed back to 6 N. In these experiments, the operating forces can be kept and adjusted accurately with the adaptation to various abdominal contours. During the process of the force change, a rise time (${t_r}$) and fall time (${t_f}$) exist because slider P needs to alter its location to change ${L_1}$. In this experiment, the rise time and fall time are 0.40 s and 0.48 s, respectively. This time is not constant and it depends on the operating force value and the position of the US probe. During the scanning, different positions may require various operating forces, and thus the operating force can be adjusted as the US probe moves from one position to another. Moreover, even for the same position, the rise and fall time is within 0.5 s, which is considered acceptable clinically for routine screening. This time delay is mainly affected by the moving speed of slider P, and it can be decreased by using a motor and gearbox with a higher output speed. This method would be feasible for the US inspections that have higher requirements for the time delay.

### E. Phantom Experiment

**Fig. 12.**
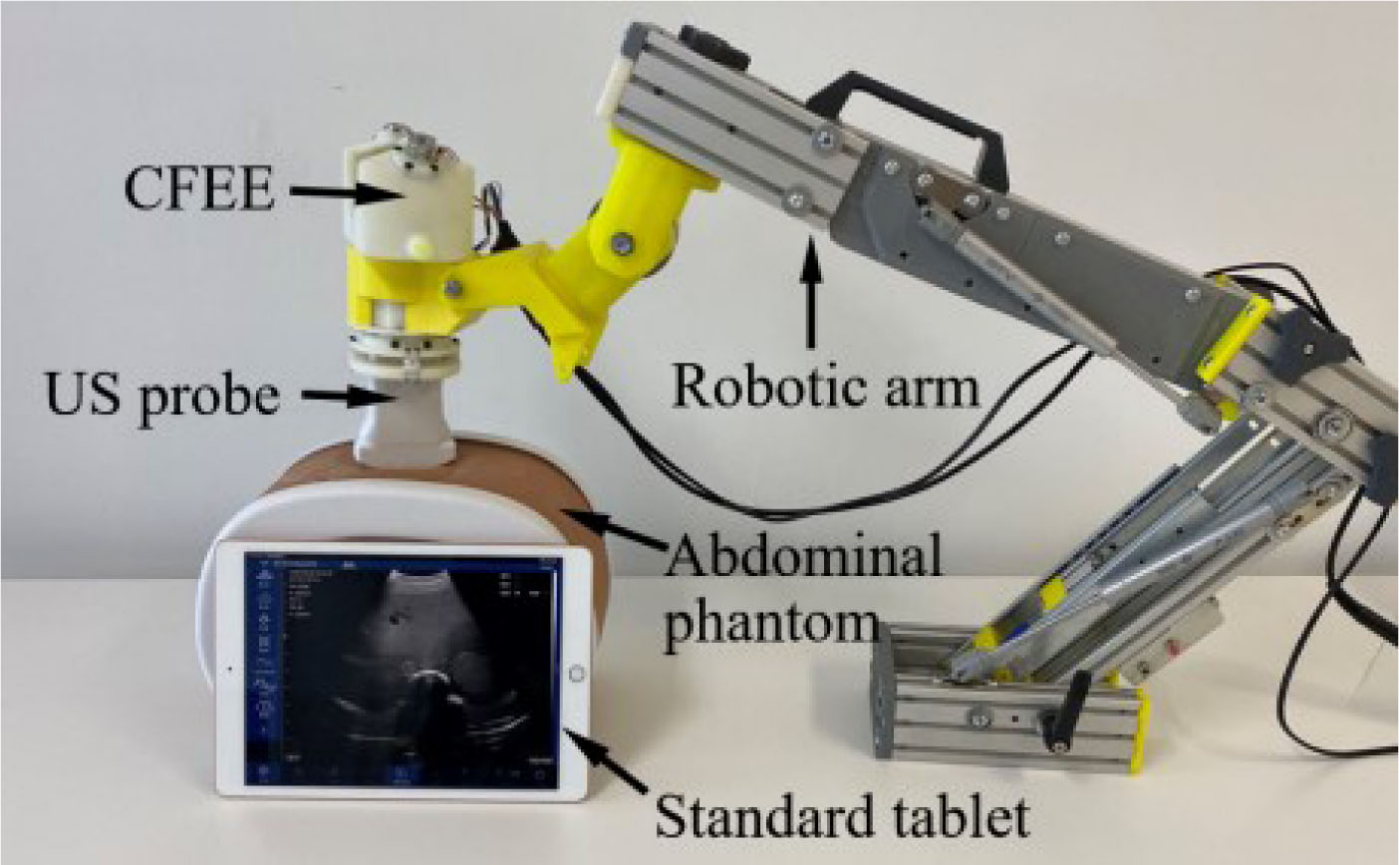
Experimental set-up for the phantom experiment.

An abdominal US phantom (057A, Computerized Imaging Reference Systems, Inc., USA) is used to evaluate the imaging capabilities of a US probe guided by the proposed CFEE. As shown in Fig. 12, the CFEE mounted on the robotic arm is equipped with a wireless US probe (CProbe, Sonostar Technologies Co., Ltd., CN). The US probe is moved in the region of interest on the abdominal phantom. Images acquired by the US probe were transmitted to a standard tablet (iPad) and displayed on its screen. Image acquisition operations were performed by manual operation and by using the CFEE, respectively. The operating force for the CFEE was set as 10N.

**Fig. 13.**
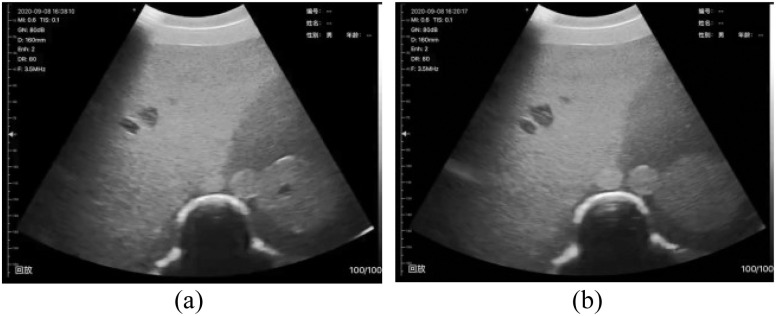
(a) US images acquired by CFEE operation; (b) US images acquired by manual operation.

Fig. 13 shows the US images acquired through CFEE and manual operation. The spine and kidney structures of this abdominal US phantom are clearly visible in both the CFEE and manual operation. Moreover, the contrast in the CFEE-acquired images is similar to the one in the manually-obtained images.

## IV. Conclusion

In this letter, a CFEE was proposed to provide a constant operating force over a wide range and adjust the operating force online to adapt to various conditions and the contours of scanned areas. The performance was preliminarily verified through simulations and experiments. The proposed CFEE has a simple structure and low cost and can be easily applied to other medical applications. Robotic ultrasonography with the proposed CFEE would have the following advantages: high-quality images, comfort and safe operation for patients, and high work efficiency and low workload for sonographers. Moreover, this method could be extended to other applications with constant- force operation. In our future work, we will verify the feasibility of the force measurement unit by conducting healthy volunteer studies.
